# Apparent bilateral aldosterone suppression during adrenal venous sampling associated with preferential drainage into the left inferior phrenic vein

**DOI:** 10.1530/EDM-26-0013

**Published:** 2026-10-03

**Authors:** Tomoko Nishigaki, Masaaki Yamamoto, Yuichi Higuchi, Kei Yoshino, Hironori Bando, Keigo Matsushiro, Tomoyuki Gentsu, Keitaro Sofue, Maki Kanzawa, Wataru Ogawa, Hidenori Fukuoka

**Affiliations:** ^1^ Division of Diabetes and Endocrinology, Department of Internal Medicine, Kobe University Hospital, Kobe, Japan; ^2^ Division of Diabetes and Endocrinology, Department of Internal Medicine, Kobe University Graduate School of Medicine, Kobe, Japan; ^3^ Department of Diagnostic and Interventional Radiology, Kobe University Hospital, Kobe, Japan; ^4^ Department of Diagnostic Pathology, Kobe University Hospital, Kobe, Japan

**Keywords:** adrenal, primary aldosteronism, bilateral aldosterone suppression, adrenal venous sampling

## Abstract

**Summary:**

A 54-year-old woman with primary aldosteronism caused by left adrenal adenoma underwent adrenal venous sampling (AVS) for subtype classification and unexpectedly exhibited bilateral aldosterone suppression (BAS) despite adequate selectivity. BAS on AVS is a recognized diagnostic pitfall that can obscure surgically curable unilateral diseases, particularly left-sided cases. In this case, venous anatomy was carefully reassessed considering the inconclusive AVS findings. Although adrenal arteriography is infrequently used in the diagnostic workup of primary aldosteronism, it was incorporated as an adjunct to assess venous anatomy. No additional drainage route was identified on the obtained angiographic images, and the cranial and medial superficial location of the tumor in close proximity to the left inferior phrenic vein raised the suspicion of preferential drainage into this vessel. Although the complete tumor-drainage pathway was not directly visualized, targeted venous sampling based on this anatomical hypothesis enabled accurate lateralization of a unilateral disease. This case underscores interpreting BAS within the context of venous anatomy, particularly on the left side, rather than as definitive evidence of bilateral primary aldosteronism.

**Learning points:**

- Bilateral aldosterone suppression (BAS) on adrenal venous sampling can occur in unilateral primary aldosteronism and may reflect aberrant venous drainage; therefore, it should not be interpreted as definitive evidence of bilateral disease.
- Aberrant venous drainage, such as preferential distal drainage into the left inferior phrenic vein, should be considered when BAS occurs despite adequate selectivity.
- Careful reassessment of venous anatomy and anatomically guided targeted venous sampling can facilitate accurate lateralization and prevent misclassification.

## Background

Primary aldosteronism (PA) is a common and potentially curable cause of secondary hypertension. Accurate subtype classification is essential for determining appropriate treatment strategies (1). Adrenal venous sampling (AVS) remains the gold standard for distinguishing unilateral from bilateral disease, particularly when imaging findings are inconclusive or discordant with the clinical features (2). However, atypical venous drainage can hamper AVS interpretation. One such diagnostic pitfall is bilateral aldosterone suppression (BAS), in which aldosterone concentrations appear to be suppressed in both adrenal veins despite adequate selectivity. BAS has been reported in patients with unilateral PA and may be misclassified as a bilateral disease if not carefully evaluated (3). This phenomenon has been reported in left-sided cases where anatomical variations and alternative venous drainage pathways are relatively common (4). Previous case reports have demonstrated that aberrant venous drainage, such as drainage into the renal capsular vein or left inferior phrenic vein (LIPV), can account for apparent BAS by diverting aldosterone-rich blood from the sampled adrenal vein (5, 6). Herein, we report a left-sided aldosterone-producing adenoma presenting with BAS on AVS, in which careful anatomical evaluation and targeted venous sampling enabled accurate lateralization.

## Case presentation

A 54-year-old woman with long-standing hypertension and recent hypokalemia was referred for evaluation of suspected PA. She was diagnosed with hypertension at the age of 42 years. At age 44, her aldosterone-to-renin ratio (ARR) exceeded 200 while she was taking an angiotensin receptor blocker, but no definitive workup was performed. At age 52, recurrent hypokalemia prompted outpatient testing, which revealed a plasma renin activity (PRA) of 0.4 ng/mL/h, a plasma aldosterone concentration (PAC) of 102.1 pg/mL, and an ARR of 405, raising suspicion for PA. She had no history of diabetes mellitus, dyslipidemia, or chronic kidney disease. Family history included maternal hypertension and malignancies in grandparents (colon and liver cancer). She was a nonsmoker and nonalcoholic. At referral, her medications included nifedipine 40 mg/day and oral potassium chloride 3,600 mg/day. At admission, her blood pressure was 121/77 mmHg, and serum potassium was 3.7 mEq/L despite potassium supplementation. Serum creatinine was 0.56 mg/dL, with an estimated glomerular filtration rate of 86 mL/min/1.73 m^2^; urinary protein was negative; and the urinary albumin-to-creatinine ratio was 28.21 mg/g creatinine. Echocardiography showed mild concentric left ventricular hypertrophy. She had no history of atrial fibrillation, stroke, coronary artery disease, or heart failure, and electrocardiography showed sinus rhythm.

## Investigation

Laboratory data revealed a high PAC (480.0 pg/mL, measured by radioimmunoassay, normal range (NR): 4.0–82.1 pg/mL), a low PRA (0.3 ng/mL/h; NR: 0.2–2.3 ng/mL/h), and a markedly elevated ARR (1,600; screening positive, ARR ≥ 200 and PAC ≥ 60 pg/mL), fulfilling the screening criteria for PA. A captopril challenge test demonstrated a persistently suppressed PRA (0.3–0.4 ng/mL/h) with a sustained elevation of PAC (354.8–376.8 pg/mL), resulting in a consistently high ARR (>900), thereby confirming autonomous aldosterone secretion. A 1-mg overnight dexamethasone suppression test revealed adequate cortisol suppression (adrenocorticotropic hormone (ACTH): 1.6 pg/mL, serum cortisol (F): 0.9 μg/dL), showing no evidence of autonomous cortisol secretion. Abdominal computed tomography (CT) revealed a 12-mm round left adrenal lesion (Fig. 1). Early-phase contrast-enhanced CT images demonstrated that the tumor was located in the cranial and medial superficial portion of the left adrenal gland. ACTH-stimulated AVS was performed. Catheter positions were confirmed by CT, and selectivity index (SI) values exceeded the prespecified threshold (>5; right adrenal vein, 22.6; left adrenal vein–LIPV common trunk, 33.3; left adrenal vein, 22.1), indicating technically successful sampling. Contrary to expectations, the PAC-to-cortisol (PAC/F) ratios in the right adrenal vein (4.0), left adrenal vein–LIPV common trunk (2.8), and left adrenal vein (3.8) were all lower than that in the IVC (35.1), indicating BAS (Fig. 2). To investigate a possible extra-adrenal source of aldosterone production, ^131^I-adosterol scintigraphy was performed under dexamethasone suppression, which revealed no additional extra-adrenal uptake. To investigate the anatomical contributors to BAS and identify possible venous drainage variations before the second AVS, selective left superior adrenal arteriography was performed. In the venous phase of the arteriogram, both the left adrenal vein–LIPV common trunk and the LIPV were visualized; notably, the cranial portion of the LIPV, corresponding to the superior aspect of the adrenal gland, opacified earlier and more intensely than the common trunk. No additional drainage route was identified on the obtained angiographic images (Fig. 3A). Based on these findings, retrograde venography from the left common trunk was subsequently performed, which opacified a markedly developed LIPV coursing adjacent to the tumor (Fig. 3B). Therefore, a second AVS was undertaken after intravenous administration of 250 μg cosyntropin, with anatomically targeted sampling at six sites (a–f; Fig. 3B). All sampling sites met the prespecified selectivity criterion (SI > 5), with SI values ranging from 6.7 to 36.0 (Fig. 3C). The PAC/F ratio was markedly elevated in the intermediate and distal LIPV (sites d and e: 91.3 and 745.9, respectively), compared with 1.9 in the right adrenal vein (site f). The corresponding lateralization indices were 48.1 and 392.6, respectively, and the right-sided contralateral suppression index was 0.063. These findings established left-sided lateralization and supported left adrenalectomy. Based on the angiographic and biochemical findings, preferential tumor drainage into the distal LIPV was hypothesized, although the complete tumor-drainage pathway was not directly visualized (Fig. 3D).

**Figure 1:**
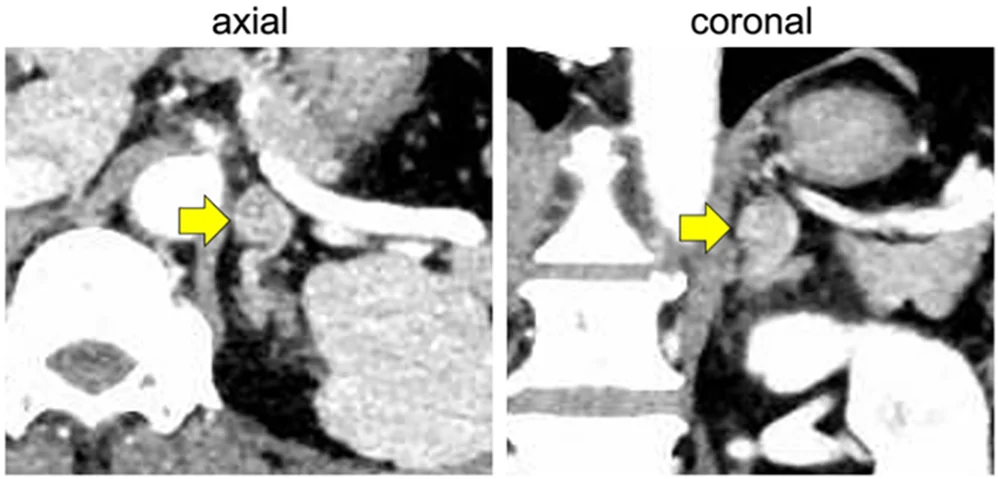
Abdominal computed tomography (CT) findings. Early-phase contrast-enhanced CT images in the axial (A) and coronal (B) planes showing a 12-mm round tumor in the cranial and medial superficial portion of the left adrenal gland. The arrows indicate the tumor.

**Figure 2:**
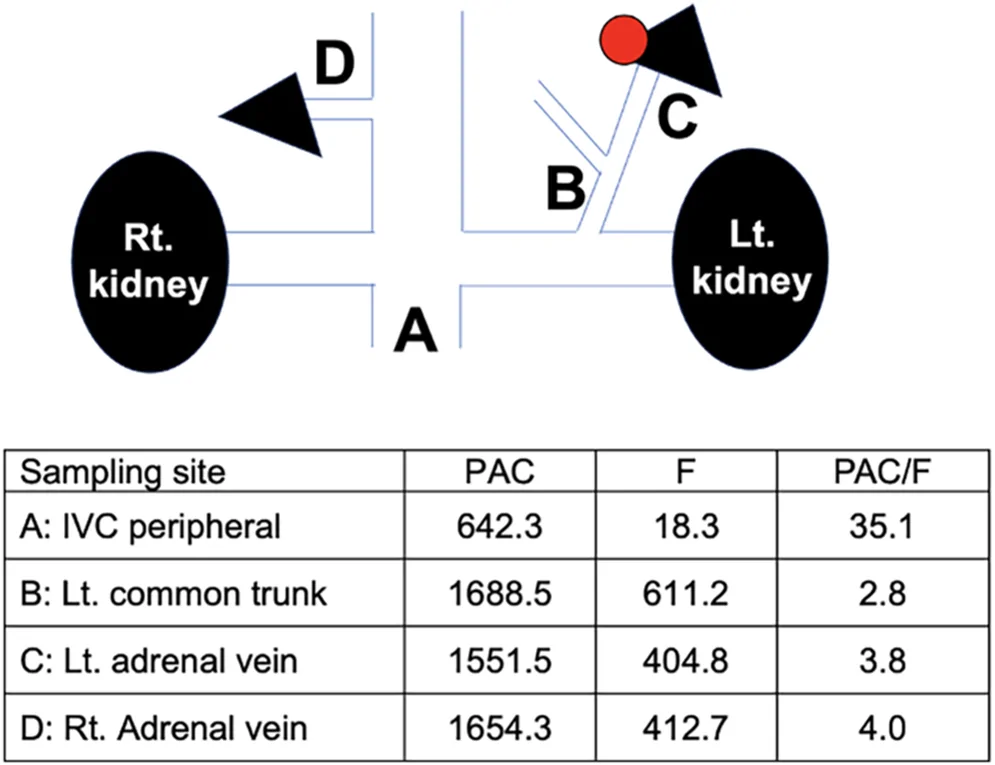
First ACTH-stimulated adrenal venous sampling, demonstrating apparent bilateral aldosterone suppression. Despite successful cannulation, as indicated by adequate selectivity indices (right adrenal vein, 22.6; left adrenal vein–LIPV common trunk, 33.3; left adrenal vein, 22.1), PAC-to-cortisol (PAC/F) ratios in the right adrenal vein (4.0), left adrenal vein–LIPV common trunk (2.8), and left adrenal vein (3.8) were all lower than that in the inferior vena cava (IVC, 35.1), consistent with apparent bilateral aldosterone suppression. ACTH, adrenocorticotropic hormone; IVC, inferior vena cava; LIPV, left inferior phrenic vein; PAC, plasma aldosterone concentration; F, cortisol.

**Figure 3:**
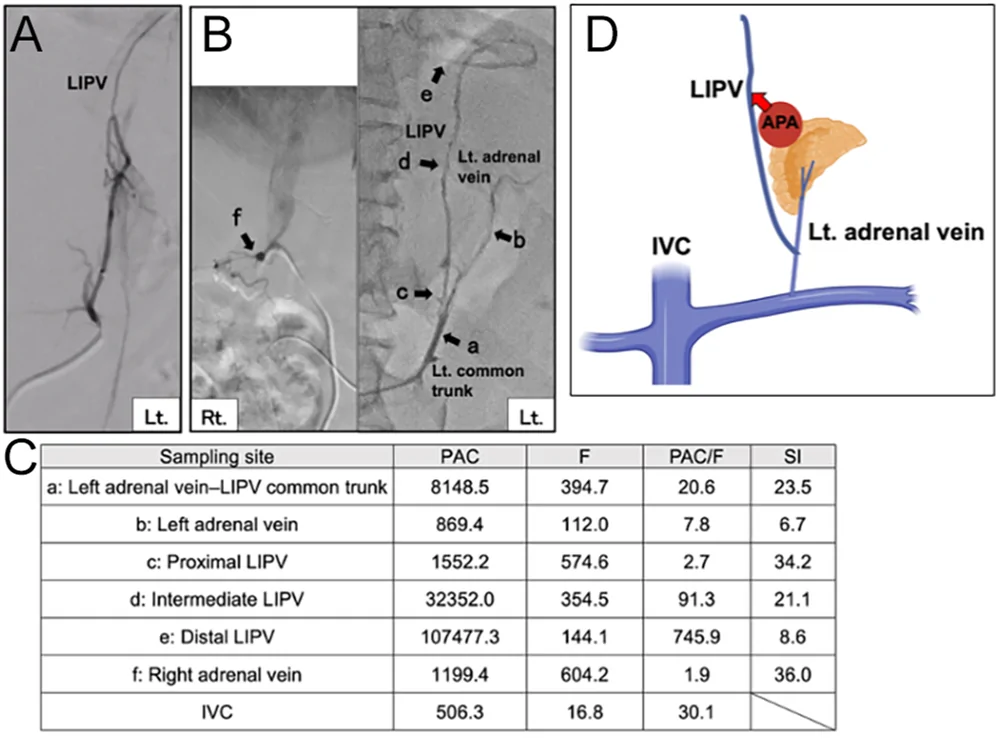
Adjunctive angiographic evaluation and anatomically targeted repeat adrenal venous sampling. (A) Venous-phase image of selective left superior adrenal arteriography demonstrating opacification of the left inferior phrenic vein (LIPV). (B) Venographic images during repeat AVS, showing the anatomically targeted sampling sites. Sampling sites are given in Panel C. (C) Biochemical results of the targeted sampling, including plasma aldosterone concentration (PAC), cortisol (F), PAC/F ratio, and selectivity index (SI). (D) Schematic illustration of the hypothesized venous drainage pattern. The red arrow indicates the proposed preferential drainage of tumor effluent into the distal LIPV; the complete tumor-drainage pathway was not directly visualized. AVS, adrenal venous sampling; APA, aldosterone-producing adenoma; IVC, inferior vena cava.

## Treatment

The patient underwent a laparoscopic left adrenalectomy without complications. On gross examination, the resected specimen revealed a well-circumscribed tan-yellow adrenocortical nodule measuring 12 mm, protruding medially from the adrenal surface. Histopathological examination confirmed an adrenocortical adenoma without malignant features, and immunohistochemistry for aldosterone synthase (CYP11B2) showed diffuse positivity in the tumor cells, confirming an aldosterone-producing adenoma (Fig. 4).

**Figure 4:**
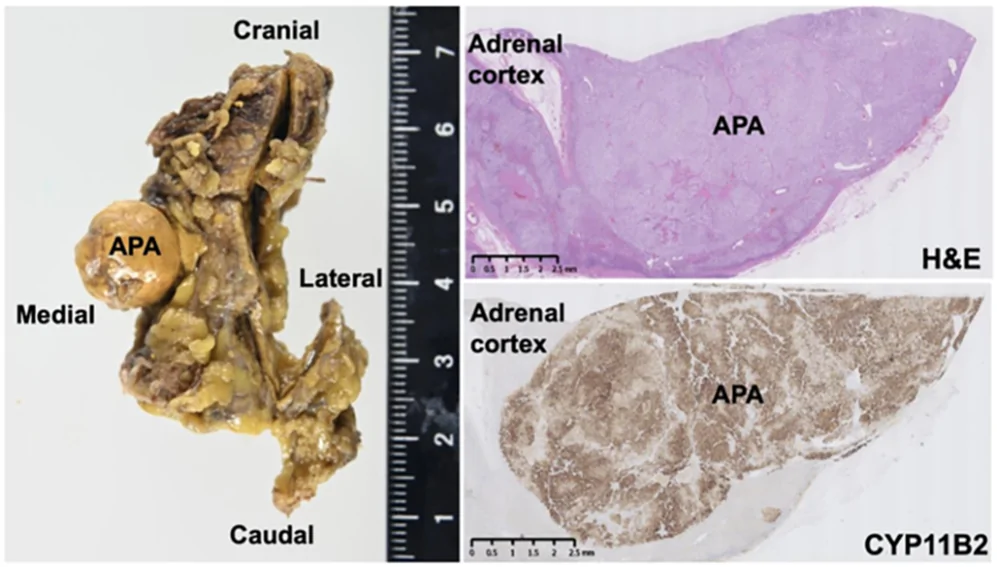
Macroscopic and histopathological findings of the left adrenal tumor. Gross examination of the resected adrenal gland showing a well-circumscribed tumor protruding medially from the adrenal surface; cranial–caudal and medial–lateral orientations are indicated. Hematoxylin–eosin staining demonstrated an adrenocortical adenoma, and immunohistochemical staining for aldosterone synthase (CYP11B2) was positive in tumor cells, confirming an aldosterone-producing adenoma. APA, aldosterone-producing adenoma; H&E, hematoxylin and eosin.

## Outcome and follow-up

At approximately 3 months after surgery, serum potassium had normalized to 4.1 mEq/L without potassium supplementation, PRA had increased to 1.4 ng/mL/h, and PAC had decreased to 50.2 pg/mL, yielding an ARR of 35.8. These findings met the biochemical criteria for complete success defined by the Primary Aldosteronism Surgical Outcome (PASO) consensus (7). Her blood pressure was 132/80 mmHg while receiving amlodipine 5 mg/day. At the latest in-person follow-up approximately 5 months after surgery, her blood pressure was 110/78 mmHg on amlodipine 5 mg/day, and serum potassium remained normal at 4.6 mEq/L without supplementation. The clinical outcome at that time was consistent with partial clinical success.

## Discussion

BAS on AVS represents a diagnostic pitfall that may obscure surgically curable unilateral PA, particularly in left-sided cases. Aberrant venous drainage, in which aldosterone-rich effluent bypasses the central adrenal vein and drains into alternative pathways, such as the renal capsular vein or duplicated adrenal veins, has been implicated in left-sided BAS (5, 6). In such settings, conventional central adrenal vein sampling may underestimate aldosterone secretion, despite successful cannulation. Apparent BAS can also occur in right-sided disease, for example, when superselective right adrenal vein sampling obtains blood from a tributary that does not receive aldosterone-rich tumor effluent (8).

Several reports of left-sided cases support this mechanism. Hirose *et al.* demonstrated markedly elevated aldosterone concentrations in the renal capsular vein draining a left aldosterone-producing adenoma despite apparent BAS on standard AVS (5). Tannai *et al.* reported a left-sided case in which aldosterone excess was identified only by sampling an independent LIPV, demonstrating that preferential drainage into the LIPV can cause BAS (6). Collectively, these cases indicate that left-sided BAS frequently reflects the diversion of aldosterone-rich blood away from the sampled adrenal vein rather than true BAS. In contrast to these previous reports, the present case illustrates the sequential use of adrenal arteriography, retrograde venography, and anatomically targeted repeat sampling after initially inconclusive AVS. This approach identified the sampling sites that demonstrated left-sided aldosterone excess and provided the basis for surgical treatment.

Although adrenal arteriography is occasionally included in the diagnostic workup of PA, it was incorporated in the present case as an adjunct to assess venous anatomy in the setting of inconclusive AVS findings. No additional drainage route was identified on the obtained angiographic images, and the cranial and medial superficial location of the tumor in close proximity to the LIPV supported the hypothesis of preferential drainage into this vessel and guided targeted venous sampling, as reported in selected cases using adrenal arteriography (9). Whether the intraglandular location of an aldosterone-producing adenoma is systematically associated with the occurrence of BAS remains unclear. In the present case, the spatial relationship between the cranial and medial superficial tumor and the adjacent LIPV made preferential drainage into this vessel anatomically plausible, but this case-specific finding does not establish a general association between tumor location and BAS.

Large retrospective datasets from Japan support this diagnostic approach. In a multicenter analysis, BAS was observed in a substantial proportion of patients undergoing AVS. Although its frequency decreased after ACTH stimulation, a subset of cases persisted despite adequate selectivity. Notably, BAS can often be resolved when alternative drainage routes or additional sampling sites are considered, indicating that BAS should prompt anatomical reassessment rather than immediate classification as a bilateral disease (3).

In this case, left-sided BAS was interpreted based on previous case reports and retrospective data. Although the complete tumor-drainage pathway into the LIPV was not directly visualized, the angiographic findings and markedly elevated PAC/F ratios in targeted LIPV samples strongly supported this anatomical hypothesis. A careful evaluation of venous anatomy, combined with targeted sampling based on a defined anatomical hypothesis, allows for accurate lateralization of unilateral disease. This case underscores that, particularly on the left side, BAS should prompt consideration of venous drainage patterns rather than be taken as definitive evidence of bilateral PA.

## Declaration of interest

The authors declare that there is no conflict of interest that could be perceived as prejudicing the impartiality of the work reported.

## Funding

This work was partially supported by a Grant-in-Aid for Scientific Research from the Japan Society for the Promotion of Science (KAKENHI, grant number 23K08008 (MY)).

## Patient consent

Written informed consent for publication of their clinical details and clinical images was obtained from the patient.

## Author contribution statement

TN was responsible for patient management, data collection, and manuscript drafting. YH and KY contributed to clinical diagnosis and treatment; KM, TG, and KS performed catheter intervention and radiological diagnosis; and MK was involved in pathological diagnosis. HB, WO, and HF contributed to the critical revision of the article for important intellectual content. MY conceptualized, wrote, reviewed, and supervised the study. All authors reviewed and approved the final version of the manuscript for publication.
